# Mordred: a molecular descriptor calculator

**DOI:** 10.1186/s13321-018-0258-y

**Published:** 2018-02-06

**Authors:** Hirotomo Moriwaki, Yu-Shi Tian, Norihito Kawashita, Tatsuya Takagi

**Affiliations:** 1 Graduate School of Pharmaceutical Sciences, Osaka University, 1-6 Yamadaoka, Suita City, Osaka 565-0871 Japan; 2 Faculty of Sciences and Engineering, Kindai University, 3-4-1 Kowakae, Higashiosaka City, Osaka 577-8502 Japan

**Keywords:** Molecular descriptor, QSPR, Cheminformatics, Calculation software, Python

## Abstract

Molecular descriptors are widely employed to present molecular characteristics in cheminformatics. Various molecular-descriptor-calculation software programs have been developed. However, users of those programs must contend with several issues, including software bugs, insufficient update frequencies, and software licensing constraints. To address these issues, we propose Mordred, a developed descriptor-calculation software application that can calculate more than 1800 two- and three-dimensional descriptors. It is freely available via GitHub. Mordred can be easily installed and used in the command line interface, as a web application, or as a high-flexibility Python package on all major platforms (Windows, Linux, and macOS). Performance benchmark results show that Mordred is at least twice as fast as the well-known PaDEL-Descriptor and it can calculate descriptors for large molecules, which cannot be accomplished by other software. Owing to its good performance, convenience, number of descriptors, and a lax licensing constraint, Mordred is a promising choice of molecular descriptor calculation software that can be utilized for cheminformatics studies, such as those on quantitative structure–property relationships.

**Electronic supplementary material:**

The online version of this article (10.1186/s13321-018-0258-y) contains supplementary material, which is available to authorized users.

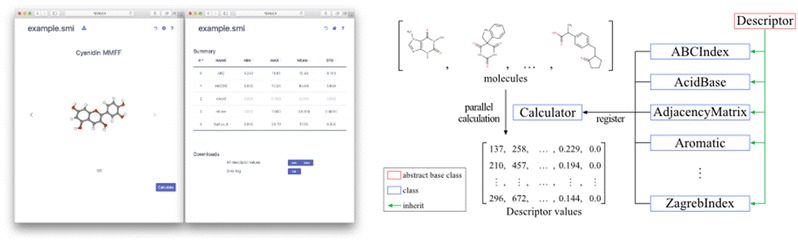

## Background

A molecular descriptor is defined as the “final result of a logical and mathematical procedure, which transforms chemical information encoded within a symbolic representation of a molecule into a useful number or the result of some standardized experiment” [1]. Many types of molecular descriptors have been developed, such as the number of carbon atoms; molecular weight; predictive values of LogP (XLogP [2], ALogP [3], etc.); properties calculated from two-dimensional (2D) structures (e.g., Eccentric Connectivity Index [4]) and three-dimensional (3D) structures (e.g., charged partial surface area (CPSA) [5]); and properties based on quantum mechanics (orbital energies of highest occupied molecular orbital (HOMO), lowest unoccupied molecular orbital (LUMO), etc.). Quantitative structure–property relationship (QSPR) models frequently use molecular descriptors. They are studied to predict the activity, toxicity, and other properties resulting from the chemical structures of compounds.

The steps in a general procedure of QSPR model construction using molecular descriptors are outlined below.

1. Split the dataset into training and test datasets for evaluating the predicted performance of the model.
2. Calculate numerous molecular descriptors of each compound in the datasets.
3. Construct a reliable model of the training dataset to predict the target activity or property from these calculated descriptors using classification or regression methods (e.g., multiple regression analysis, partial least squares regression, support vector machine (SVM), and random forest).
4. Evaluate the performance of the constructed model by predicting the target activities of the compounds in the test dataset that are not used for model construction.

Although both open-source and proprietary software have been developed for calculating molecular descriptors—such as PaDEL-Descriptor [6], BlueDesc [7], ChemoPy [8], PyDPI [9], Rcpi [10], cinfony [11], and Dragon [12]—they each have advantages and disadvantages (Table 1). The freely available PaDEL-Descriptor can calculate 1875 molecular descriptors and fingerprints. It provides several interfaces (e.g., a graphical user interface (GUI), command line interface (CLI), Konstanz Information Miner (KNIME) [13], and Rapid Miner [14]), and it has been cited by other papers more than 300 times. Moreover, it can calculate descriptors in parallel. Thus, these advantages make it one of the best choices among open-source molecular-descriptor calculators. However, we determined that it has several defects (Table 2), which implies that careful verifications and modifications are required when using it.

**Table 1:** Comparison features of major descriptor calculation software

	Mordred	PaDEL-Descriptor	BlueDesc	ChemoPy	PyDPI	Rcpi	Cinfony	Dragon
Number of descriptors	1825	1875	174	1135	615	307	–^a^	5270
Citation count^b^	–	598	–	48	17	21	38	148
Library	Python2/3	–	–	Python2	Python2	R	Python2/3	–
Parallel computation	✓	✓	–	–	–	–	–	–
GUI	–	✓	–	–	–	–	–	✓
CLI	✓	✓	✓	–	–	–	–	✓
KNIME	–	✓	–	–	–	–	–	✓
RapidMiner	–	✓	–	–	–	–	–	–
Web Interface	✓	–	–	–	–	–	–	✓^c^
Last release	2018/1/20	2014/7/21	2008/10/3	2013/2/1	2015/11/10	2017/11/18	2015/8/1	?^d^
License	BSD-3-Clause	^e^	GPL	GPL	GPL	Artistic license	BSD-2-clause/GPLv2/GPLv3	Proprietary
Source code distribution	Github	Official site	Official site	Google code	pypi	github	github	–
Other advantages			Easy to use with libSVM		Can also calculate protein descriptor	Can also calculate protein descriptor		Include analysis tool
Other disadvantages		Some bugs are founded	No configurable options				Require many manually installed dependencies	Payware

^a^Depends on backends

^b^Citation counts on Google Scholar (accessed on 2018/01/16)

^c^Provided by e-Dragon, which uses the old version of Dragon

^d^Unknown; however, Dragon is being actively developed

^e^“This software is free for all (e.g. personal, academic, non-profit, non-commercial, government, commercial, etc.) to use.” (http://yapcwsoft.com/dd/padeldescriptor, accessed on 2018/01/16)

**Table 2:** Defects identified in the descriptor calculation software

Software	Details
CDK	Theoretically, the roto-translation of a molecule should not change any molecular property. However, TPSA and LengthOverBreadth descriptors resulted in different values of molecules before and after roto-translation transformation
	The value of ChiPathCluster is invalid because the patterns are not adequate (fixed in the latest version of CDK)
PaDEL	Several molecules (e.g., Cyanidin) resulted in invalid values in many descriptors (e.g., nH (hydrogen count) returned 12) when using the default configuration owing to a bug in the aromaticity detecting procedure and/or 3D conformer generator of PaDEL-Descriptor. This caused breakage of an aromatic ring and attachment to an invalid hydrogen
	Some descriptors use the log sum exponential (LSE) function (${\text{LSE}}_{1}$ below), which is prone to arithmetic overflow or underflow. The LSE trick (${\text{LSE}}_{2}$ below) should be used to avoid this issue ${\text{LSE}}_{1} \left( {x_{1} ,x_{2} , \ldots , x_{n} } \right) = { \log }\left( {\mathop \sum \limits_{i = 1}^{n} { \exp }\left( {x_{i} } \right)} \right)$ $$\begin{array}{*{20}c} {{\text{LSE}}_{2} \left( {x_{1} ,x_{2} , \ldots , x_{n} } \right) = x^{ *} + \log \left( {\mathop \sum \limits_{i = 1}^{n} \exp \left( {x_{i} - x^{*} } \right)} \right)} \\ {x^{ *} = \hbox{max} \left( {x_{1} ,x_{2} , \ldots ,x_{n} } \right)} \\ \end{array}$$
	In the constitutional descriptor, discrepancies might be induced in the algorithm implementation owing to incorrect code reuse
ChemoPy	Cannot calculate exact values of modified Zagreb index 2

Meanwhile, BlueDesc is a simple software program for calculating descriptors. Because BlueDesc can output the results in libSVM input file format, users can easily construct SVM models. However, BlueDesc can calculate only 174 descriptors, and it has no configurable options. ChemoPy, a free software environment that calculates both 2D and 3D descriptors, can calculate 1135 descriptors. ChemoPy is available as a Python package and is convenient for constructing models using Python machine-learning packages. However, it can be difficult to employ it by non-Python users who are not familiar with the construction of the Python interface. (As described later, this disadvantage can be overcome by using several web interfaces that incorporate ChemoPy.) To date, ChemoPy only supports Python 2.

Furthermore, PyDPI can calculate small molecule descriptors as well as protein descriptors. However, it has advantages and limitations that are similar to those of ChemoPy. In addition, Rcpi (the protein and small molecule descriptor calculation package for R [15]) is the only R package to calculate small molecular descriptors and protein descriptors. It can calculate only 307 small molecular descriptors. Cinfony is another Python package. It is a Python wrapper of numerous other libraries (e.g., Open Babel [16], RDKit [17], Chemistry Development Kit (CDK) [18], Indigo [19], JChem [20], and OPSIN [21]). Thus, it requires many manually installed dependencies, which is a complicated process. Moreover, a new version has not been released since December 2012 [22]. Dragon is another widely used application software that is used to calculate molecular descriptors. It can calculate numerous descriptors, and it has several interfaces, such as a GUI, CLI, web (e-Dragon [23], which is based on an older free version of Dragon), and KNIME. However, it is proprietary shareware; its source code is not open, and it is not easy to publish constructed quantitative structure–activity relationship (QSAR) models, such as Ecological Structure Activity Relationships (ECOSAR) [24], on account of licensing issues. Furthermore, some descriptor calculation programs do not have a user-friendly interface and/or it is difficult to set up the environment. Thus, several web-based descriptor calculation interfaces have been developed, such as ChemDes [25] and BioTriangle [26]. ChemDes can calculate all descriptors that can be calculated by ChemoPy, CDK, RDKit, Open Babel, BlueDesc, and PaDEL. Moreover, BioTriangle can manipulate not only small molecules, but also nucleic acid and protein. Because users do not need to install any software except a web browser, which can be accessed from any device, web user interfaces (UIs) are convenient.

In this paper, we propose Mordred, a newly developed molecular-descriptor calculation software program. The software can calculate more than 1800 descriptors at high speed, and it can be used from the CLI and web UI, as well as with Python 2 and 3 libraries. Mordred was released under the three-clause Berkeley Software Distribution (BSD) license, which allows both commercial and non-commercial use.

## Implementation

### Motivation and Mordred concepts

Mordred was designed to be a software program that is easy to install and use, supports abundant molecular descriptors, has a high calculation speed, and includes automated tests. Molecular-descriptor calculation programs usually have many dependent software programs that must be manually installed. All direct dependent libraries in Mordred, except for RDKit and NumPy [27], are coded in pure Python (enum34, networkx, six, tqdm) to simplify the installation. RDKit and NumPy are widely used Python libraries and can be easily installed via the pre-compiled libraries distributed by the Anaconda cloud [28]. Therefore, users can install Mordred using a single command (see Code 1 below). Because Mordred can be employed as a web service or from a CLI, and with Python 2 and 3 libraries, users ranging from beginners to experts can employ it.

Preprocessing of molecules affects the descriptor values in most molecular-descriptor calculators. However, for each descriptor, Mordred automatically preprocesses molecules (adds or removes hydrogen atoms, performs Kekulization, and detects molecular aromaticity). For example, a topological index descriptor was reported by Balaban et al. [29]. along with a numerical example. To reproduce the values, the input molecule should not have explicit hydrogen atoms. Therefore, explicit hydrogen atoms are automatically removed in Mordred. This procedure ensures correctness of preprocessing, which is usually not checked in other software.
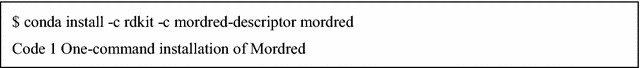


Mordred calculates more than 1800 default molecular descriptors, including all those implemented by RDKit (seven modules) and original implementations (42 modules) (Table 3). To note, PaDEL-Descriptor can calculate the largest number of descriptors among other related open-source software programs by providing 1875 molecular descriptors. The number of default descriptors calculated by Mordred is comparable, which suggests that Mordred can be used as alternative descriptor calculation software in QSAR studies. Table 2 lists the defects identified in other descriptor calculation applications. These defects were fixed in the development of Mordred. Moreover, users can employ optional descriptors by passing parameters or generating product terms of descriptors. For example, n-membered ring descriptors calculate the number of n-membered rings; n = 3 to n = 12 can be calculated by default. If the number of a larger ring, e.g., 14- to 16-membered ring macrolides, is required, the calculation can be easily achieved by passing the parameter of an n value that is larger than 12 without modifying the source code. These unique functions significantly increase the descriptor calculation abilities and distinguish Mordred from other previously developed software.

**Table 3:** List of Mordred descriptors

Descriptor name	Number of descriptors (preset)
*2D*	
ABCIndex	2
AcidBase	2
AdjacencyMatrix	13
Aromatic	2
AtomCount	16
Autocorrelation	606
BCUT^a^	24
BalabanJ^a^	1
BaryszMatrix^a^	104
BertzCT	1
BondCount	9
CarbonTypes	10
Chi	56
Constitutional	16
DetourMatrix	14
DistanceMatrix	13
EState	316
EccentricConnectivityIndex	1
ExtendedTopochemicalAtom	45
FragmentComplexity	1
Framework	1
HydrogenBond^a^	2
InformationContent	42
KappaShapeIndex	3
Lipinski	2
McGowanVolume	1
MoeType^a^	53
MolecularDistanceEdge	19
MolecularId	12
PathCount	21
Polarizability	2
RingCount	138
RotatableBond^a^	2
SLogP^a^	2
TopoPSA^a^	2
TopologicalCharge	21
TopologicalIndex	4
VdwVolumeABC	1
VertexAdjacencyInformation	1
WalkCount	21
Weight	2
WienerIndex	2
ZagrebIndex	4
*3D*	
CPSA	43
GeometricalIndex	4
GravitationalIndex	4
MoRSE	160
MomentOfInertia	3

^a^RDKit wrapper

To implement molecular descriptors that can efficiently perform very large calculations, certain calculation algorithms were improved. Accordingly, Mordred can calculate all descriptors of molecules as large as a maitotoxin (molecular weight of 3422; it is the largest non-polymer compound in nature) in an acceptable calculation time (approximately 1.2 s on an Intel^®^ Core™ i7-5930 K CPU, DDR4-2133 (quad channel) 64 GB memory machine.). On the contrary, most other software packages have difficulty in calculating complicated descriptors for large molecules; e.g., PaDEL-Descriptor falls into a missing value from a “time out” in such cases. For further information on these improvements, the Mordred documentation can be referenced.

Most descriptor calculation software programs do not include automated tests to check descriptor values in a distribution package. In Mordred, all descriptors are automatically tested to verify whether Mordred can calculate precise results using the reference values of the molecular descriptors. The reference values were collected from published studies, consensual findings of multiple descriptor-calculation programs, and results of manual checks according to the calculation algorithms published. The variations among descriptor values calculated by Mordred and other software programs are confirmed in Additional files 1 and 2. All tests are performed at each commit on the GitHub repository as well as when a new version is released. In addition, users can locally execute tests to check the installation success and calculate valid results on user platforms.

### Library design

**Fig. 1:**
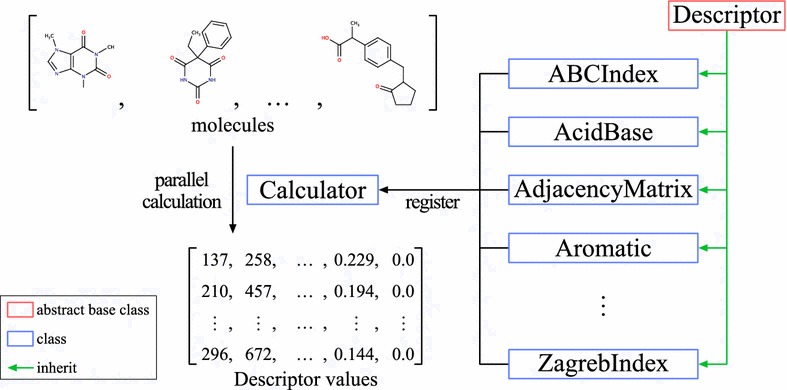
Overview of Mordred library. Mordred consists of two main classes: descriptor and calculator. Users can register descriptors on a Calculator instance. A Calculator instance can calculate descriptors in parallel

Mordred consists of two main classes: “Descriptor” and “Calculator” (Fig. 1). The algorithms of molecular descriptors are implemented in the subclass of the Descriptor class. Because there are interdependencies between the molecular-descriptor calculations, one subclass can depend on other subclasses for better efficiency in Mordred. Many descriptor-calculation algorithms provide multiple results, which complicates the treatment of descriptor values. However, each Descriptor instance provided for users returns a single value in Mordred, which makes it simple and convenient to use (Code 2). When necessary, users can employ uncommon-range descriptors by passing parameters, as shown in Code 3. Although all Mordred descriptors can be calculated by constructing descriptor instances, as shown in Code 2, this approach is not very convenient. Hence, each Descriptor class has a preset configuration, and users can obtain a set of descriptors by using this preset method (Code 4). They can be used in an instance of the Calculator class (Additional file 3).
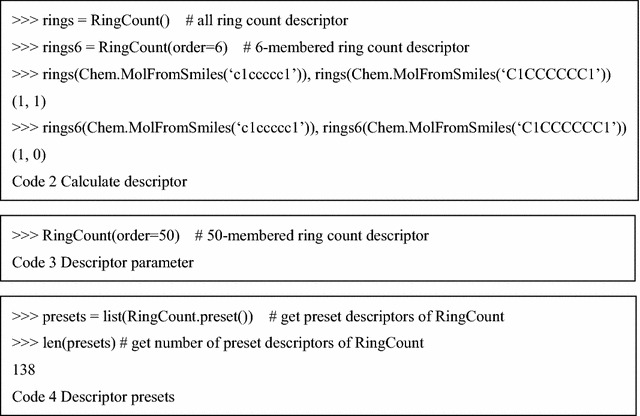

The Calculator class manages the dependencies of Descriptors, caches the results, handles errors, and enables parallel computing. Users can register the Descriptor instance (register itself), Descriptor class (preset of Descriptor), module (presets of all descriptors in the module), or a list of these in a Calculator instance. To calculate descriptors of a single molecule, an instance of Calculator can be used as a function. To calculate descriptors of multiple molecules, the map method in Calculator can be used. The map method can calculate descriptors in the CPU parallel using the multiprocessing module in the standard Python library (Code 5).
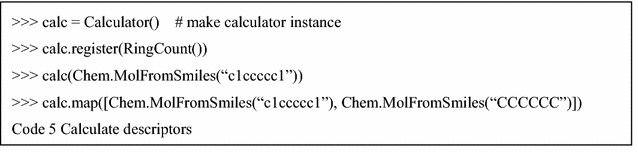


These kinds of applications have missing values, even if there are no bugs in them. For example, a part of the autocorrelation descriptor cannot be calculated for small molecules. However, unintended missing values might cause errors or result in incorrect values. Therefore, it is important to reduce the missing values caused by software bugs or calculation timeouts. In Mordred, when the result is a missing value, an instance of the subclass MissingValueBase is returned. It contains an Exception that notifies users about the issue that triggered the exception (Code 6).
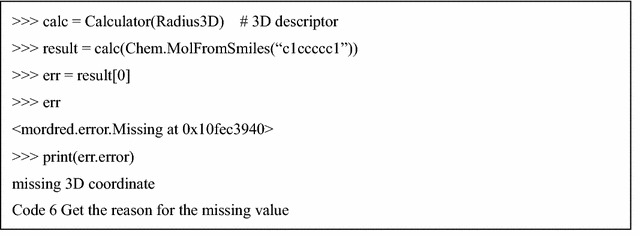


As an option, if the expression of “MissingValue” is not necessary, these missing values can be filled with “NaN” or deleted by the fill_missing or drop_missing method of the Result class. Moreover, users can obtain results as a dictionary using the asdict method of the Result class (Code 7).
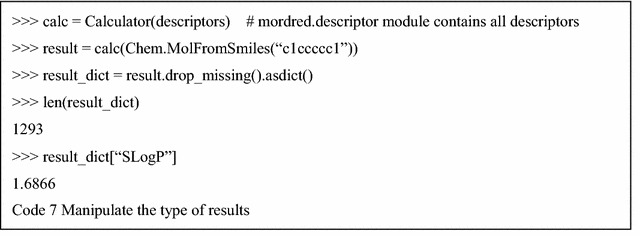


The product term is often used to consider the interaction between features in a linear model. Mordred can easily calculate the product term using the “descriptor arithmetic” feature. Instances of descriptors can be calculated by employing a unary/binary operator with other descriptors (Code 8).
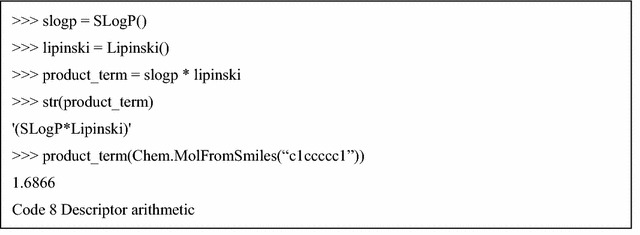


The full executable code example of Code 2–8 can be found in Additional file 4.

### Improving the algorithm

Several descriptors are algorithmically improved in Mordred. A summary of the improvements is shown in Table 4. For example, the DetourMatrix descriptor [30] requires the solving of the longest path problem between all nodes, which is an NP-hard problem. Thus, it is difficult to solve this problem efficiently. An algorithm [31] was already reported to solve this problem. However, it is not adequately efficient. The longest path problem cannot be solved using the divide and conquer method. However, it can be solved using the articulation point. The articulation point refers to the vertex, which disconnects the graph when it is removed. Chemicals generally form a sparse graph that has many articulation points. Hence, we use this property to solve DetourMatrix. First, all articulation points are found (this can be efficiently solved) and the chemical structures are split into subgraphs. Then, the longest path is searched using the depth-first search for all subgraphs (usually this can be solved because subgraphs are smaller than the original graph). Finally, the longest paths of subgraphs are merged. Other elements are filled by adding the longest paths of each subgraph.

**Table 4:** Summary of descriptor improvement

Descriptor	Summary
Chi	Depth-first search is used instead of SMARTS pattern matching
DetourMatrix	Molecular graph is divided into a small graph by articulation points
Framework	Specialize to Framework descriptor
MolecularId	Cache parts of the calculation to avoid its redundancy

**Fig. 2:**
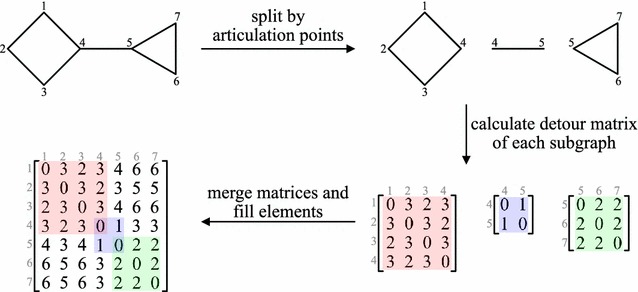
DetourMatrix algorithm. The chemical structures are split into subgraphs by all articulation points. Then, the detour matrix of each subgraph is calculated. Finally, the detour matrices of subgraphs are merged and other elements are filled

For example, to calculate the longest path from atom 1 to atom 5 shown in Fig. 2, the longest path of atom 1 to atom 4 (subgraph 1; magenta) and atom 4 to atom 5 (subgraph 2; cyan) are added. This algorithm provides a possible approach for calculating DetourMatrix for most non-polymer compounds (Fig. 1). For example, DetourMatrix of a maitotoxin can be calculated in approximately 1.2 s on an Intel^®^ Core™ i7-5930 K CPU, DDR4-2133 (quad channel) 64 GB memory machine.

The Chi descriptor is calculated by matching all Smiles Arbitrary Target Specification (SMARTS) patterns in CDK. This approach is time consuming and cannot be used to calculate the higher-order Chi descriptor. The depth-first search is used in Mordred to overcome these issues.

The Framework descriptor is calculated using the function to match the molecular framework in CDK. However, during this process, this function also performs extra calculations to solve more general cases, such as enumerating the atoms in a molecular framework. Those calculations are not necessary for the derivation of the Framework descriptor and they are removed in Mordred.

The MolecularId descriptor calls the internal pure function with the same arguments many times in CDK. The returned values of these function calls are cached to avoid same calculations in Mordred.

### Create user interface

The CLI is additionally implemented for non-Python users (Code 9). Accordingly, the entire set or subset of molecular descriptors can be calculated in CPU parallel computing. The results are provided in CSV format.
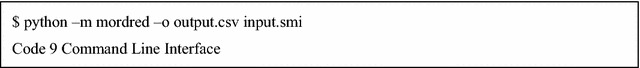


**Fig. 3:**
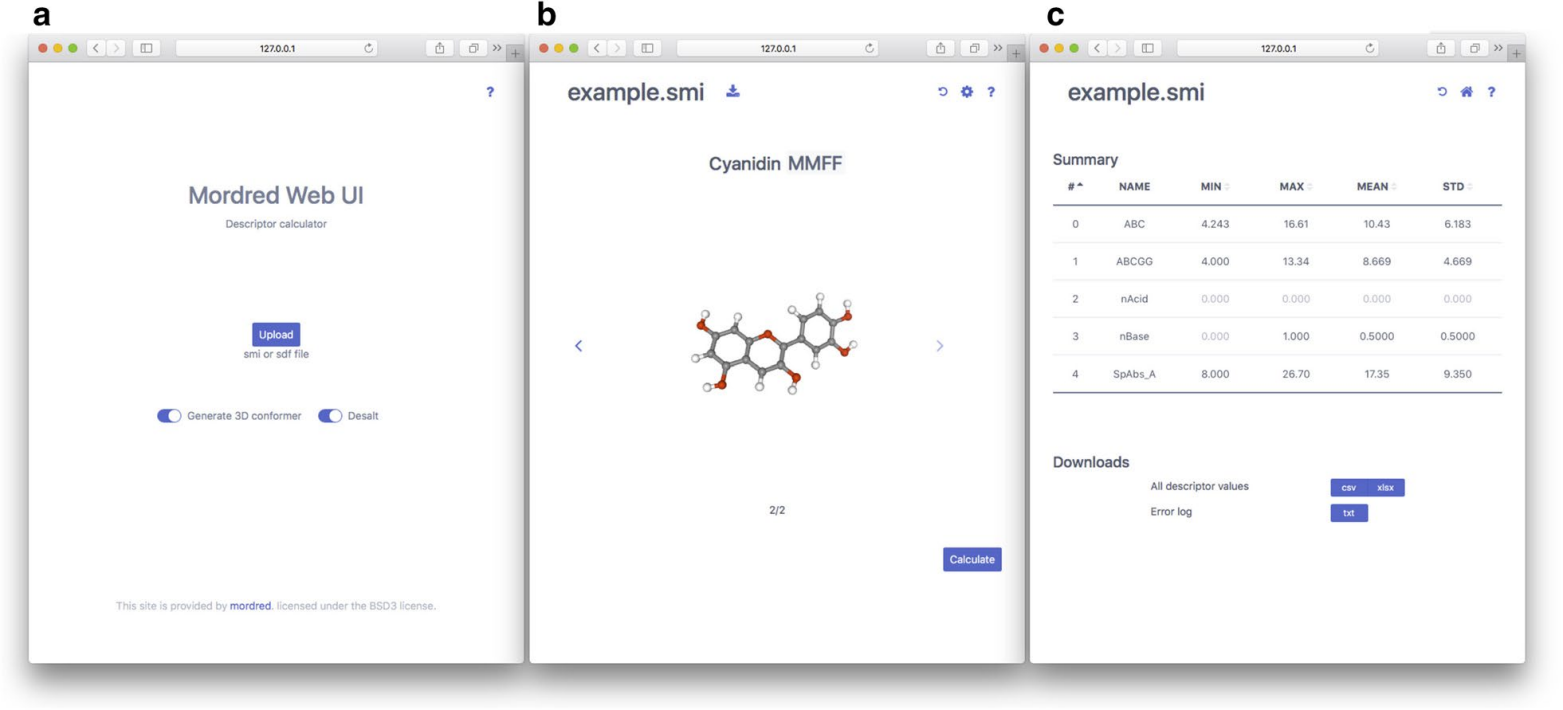
Web interface. **a** Top page of the web interface to upload a structure file,** b** preview page to check the conformation of compounds,** c** result page to confirm the descriptive statistics value of calculated descriptors and download the results

Mordred is also available as a web application (Fig. 3). The web interface is available when the Mordred web module is executed (to this end, the Mordred web package is required; the method to install this package is explained in the GitHub repository). The web interface enables beginners to easily calculate descriptors and study QSAR. In addition, Mordred can be installed on a server by an administrator. In this case, users can calculate descriptors using resources of the server simply by accessing the web page.

The web interface can work with a multi-core processor. Submitted tasks are fairly processed. A demonstration server is available at http://mordred.phs.osaka-u.ac.jp. This demonstration page works well on most widely used web browsers with current versions, such as Safari version 10+, Google Chrome version 61+, Internet Explorer version 11+, Microsoft Edge version 40+, and Firefox version 55+. However, certain browsers on some devices with legacy graphics drivers may not support this page, in which case another browser should be considered.

The usage of this demonstration page is quite simple. Briefly, a SMILE or SDF file containing single or multiple structures can be uploaded from the front page. Hydrogens can be automatically added when needed. “Generate 3D conformer” and “Desalt” buttons provide two preprocessing options. When the submission succeeds, the structures are displayed at the center of the screen. When multiple structures are uploaded, the structure can be shifted without refreshing the page by clicking the arrow buttons. If 3D conformers are provided or created, conformers can be scaled by using the middle button of the mouse, and they can be rotated by the left button of the mouse. The uploaded molecules can be downloaded in both SMILES and SDF formats by clicking the download button, which is on the right of the displayed file name. Atom names can be confirmed by hovering over the mouse.

A configuration button on the top right provides the selection options for the following descriptor calculations. By clicking the “calculate” button, users can obtain the values and summaries of descriptors, as well as the calculation log. To maintain server safety, the maximum molecule number that can be uploaded at one time is limited to 200 in this demonstration server. However, this limitation is not set by default when the user constructs his/her own server.

## Results and discussion

### Performance benchmark

To determine the speed of the calculation of molecular descriptors, we performed a benchmark test. All tests were performed on an Intel^®^ Core™ i7-5930 K CPU, DDR4-2133 (quad channel) 64 GB memory machine. We used entries from the KEGG-drug database [32] as the benchmark target. We obtained 3D structures from the LigandBox database [33]. Compounds in the dataset were converted from Tripos mol2 format to MDL mol format using Open Babel. Then, the compounds were split with respect to the number of atoms using RDKit to evaluate the time complexity. After these procedures, 7197 compounds remained. The frequency table of the number of atoms is shown as Table 5.

**Table 5:** Number of atoms in the benchmark dataset

Number of atoms	Compounds	Cumulative percentage
(0, 25]	917	12.74
(25, 50]	3412	60.15
(50, 75]	2180	90.44
(75, 100]	366	95.53
(100, 125]	147	97.57
(125, 150]	79	98.67
(150, 175]	37	99.18
(175, 200]	33	99.64
(200, 225]	12	99.81
(225, 250]	7	99.90
(250, 275]	2	99.93
(275, 300]	3	99.97
(300, 325]	1	99.99
(325, 350]	0	99.99
(350, 375]	1	100.00

(·,·] denotes a left-open and right-closed interval

We used libPaDEL-Descriptor, which is included in the source code of PaDEL-Descriptor, to evaluate the performance of the PaDEL-Descriptor because it excludes the extra descriptor calculation time, such as the file IO and molecular preprocessing. In addition, it evaluates the exact calculation time. First, each molecular descriptor was calculated three times to perform a just-in-time compilation. Then, each molecular descriptor was calculated ten times to evaluate the performance. Garbage collection was performed before each molecular-descriptor calculation. In the Mordred benchmark test, calculations were carried out ten times after disabling garbage collection.

The CLI benchmark test was performed to estimate real-world and multi-processor performance. We used the same machine. Up to 100 atoms compounds were used from the same dataset to avoid timeouts in PaDEL-Descriptor. We performed a benchmark test using one to six (the number of physical cores of an Intel Core i7-5930 K) threads (in PaDEL-Descriptor) or processes (in Mordred). Each benchmark was repeated ten times.

**Fig. 4:**
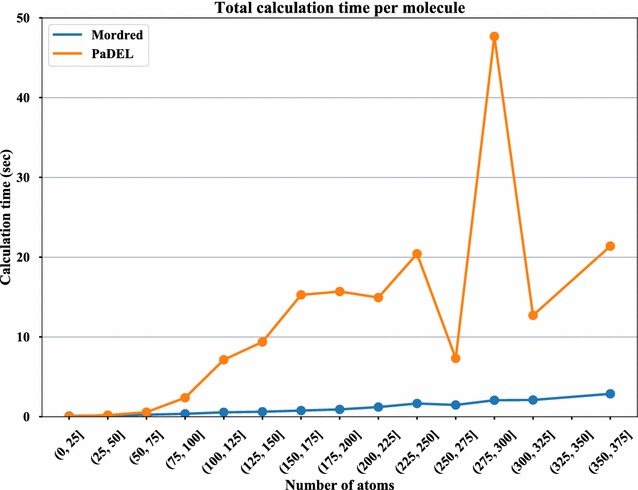
Calculation time for all descriptors. Comparison of Mordred and PaDEL-Descriptor in terms of the mean descriptor calculation time per molecule arranged by its number of atoms. The vertical axis shows the mean time of calculating all descriptors of single molecule. The horizontal axis shows class interval of number of atoms

**Fig. 5:**
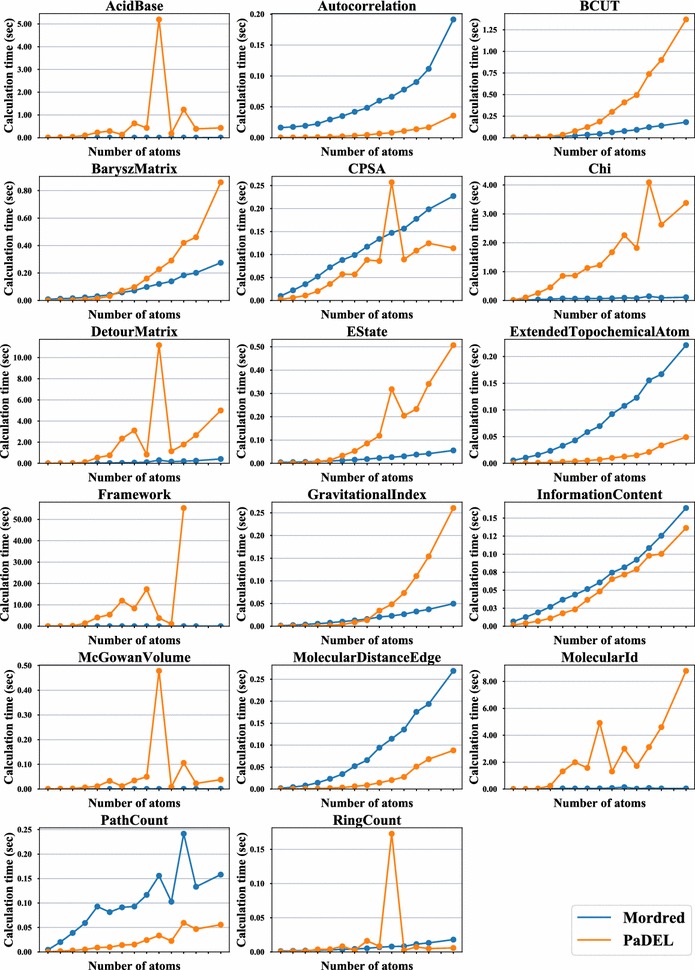
Calculation time for each descriptor. Comparison of Mordred and PaDEL-Descriptor in terms of the mean descriptor calculation time of each kind of descriptor over 0.1 s in Mordred and/or PaDEL-Descriptor. The vertical axis shows the mean time of calculating the descriptor of single molecule. The horizontal axis shows the class interval of the number of atoms

The total calculation time per molecule is shown in Fig. 4 and the calculation time of each descriptor per molecule is shown in Fig. 5. For the descriptor selection in Fig. 5, the mean calculation time of Mordred and/or PaDEL-Descriptor was over 0.1 s. DetourMatrix, Framework, and MolecularId descriptor calculation using PaDEL-Descriptor timed out when calculating descriptions for 5, 40, and 8 compounds. Figure 5 shows that AcidBase, BCUT, BaryszMatrix, Chi, DetourMatrix, Estate, and Framework are dramatically faster than the same calculations in PaDEL-Descriptor. The benchmark of Chi, DetourMatrix, Framework, and MolecularId shows the effects of improving the algorithm. On the other hand, a notable algorithm improvement is not achieved in AcidBase, BCUT, BaryszMatrix, or Estate. A performance difference of functions used in these descriptors between CDK and RDKit is apparent. PaDEL-Descriptor is faster than Mordred for many descriptor calculations, even though they both use the same algorithm. This is because Java is faster than Python for many cases. However, these descriptors can be calculated in a very short time even if Python is used. Thus, the total calculation time of Mordred, shown in Fig. 4, is much lower than that of PaDEL-Descriptor. Some descriptors, such as DetourMatrix, which result in a time-out in PaDEL-Descriptor can be calculated using Mordred. This improvement not only makes the calculation faster, but it also permits the calculation of descriptors of large molecules that cannot be calculated using other software owing to time complexity.

**Fig. 6:**
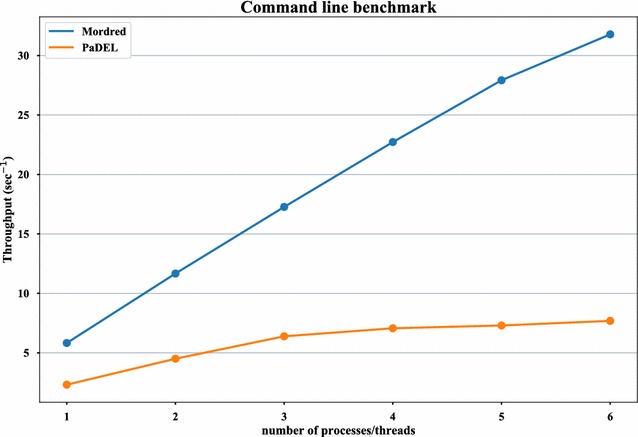
Throughput of CLI. A comparison of Mordred and PaDEL-Descriptor on the throughput of the CLI using one to six threads

The results of the CLI benchmark test are shown in Fig. 6. PaDEL-Descriptor can be scaled by up to three threads, while Mordred can be scaled by up to five threads. Moreover, Mordred is more than twofold faster than PaDEL-Descriptor for all tests, and it is four times faster for the six-thread test. These results demonstrate that Mordred is significantly more efficient than PaDEL-Descriptor.

### Comparison with other software

Mordred is superior to other software programs in several aspects. First, it is open-source software. Moreover, Mordred and all software used by it are licensed under the non-copyleft open-source license. This implies that it can be used in proprietary software. In addition, users can easily report a bug, request new features, and send patches on GitHub.

Second, it is easy to use. Mordred can be installed using only one command, whereas other Python molecular-descriptor calculation libraries (e.g., cinfony, ChemoPy) have more dependencies that require manual installation. The CLI and web interface can also be launched using a single command. Third, it has high flexibility. Mordred can calculate uncommon-range descriptors without modifying the source code. Users can create new molecular descriptors, such as the product term, by easily using the descriptor arithmetic feature. Additionally, the Mordred library can be simply accessed by using the presets in the Descriptor class. Finally, it is fast. Mordred can calculate an entire molecular descriptor twice as fast as PaDEL-Descriptor. Furthermore, the time complexity for some molecular descriptors is reduced in Mordred. As a result, it can be used for calculating the descriptors for large molecules that cannot be calculated using other software.

### Limitations

There are some potential defects in Mordred and/or its dependent libraries. We are receptive to feedback, questions, and bug reports via both email and GitHub.

## Conclusion

In this paper, we proposed one molecular descriptor calculation software named Mordred. This software is easy to install and use, generates abundant molecular descriptors in a high calculation speed. It can be installed using a single command and has a web interface. Mordred can calculate more than 1800 descriptors and calculates all descriptors in acceptable time. Our proposed software was released under a 3-clause BSD license on https://github.com/mordred-descriptor.

## Additional files

**Additional file 1.** The variations among descriptor values calculated by Mordred and other software programs.

**Additional file 2.** A confirmation of the variation among descriptor values calculated by Mordred and other software programs (PaDEL-Descriptor, chemopy and e-dragon).The results of some descriptors (e.g., 0167) are not available in all software because the descriptor of the molecule cannot be algorithmically calculated.

**Additional file 3.** All descriptor list.

**Additional file 4.** Code examples.

## Abbreviations

BSD: Berkeley Software Distribution
CDK: Chemistry Development Kit
CLI: command line interface
CPU: central processing unit
CPSA: charged partial surface area
DDR4: double data rate 4
GUI: graphical user interface
HOMO: highest occupied molecular orbital
KEGG: Kyoto Encyclopedia of Genes and Genomes
KNIME: Konstanz Information Miner
LSE: log sum exponential
LUMO: lowest unoccupied molecular orbital
NP: non-deterministic polynomial time
QSAR: quantitative structure–activity relationship
QSPR: quantitative structure–property relationship
SMARTS: smiles arbitrary target specification
SMILES: simplified molecular input line entry system
SVM: support vector machine
TPSA: topological polar surface area
