# Supplementary material for: Mordred: a molecular descriptor calculator
Source: J Cheminform. 2018 Feb 6;10:4. doi: 10.1186/s13321-018-0258-y (PMC5801138; doi:10.1186/s13321-018-0258-y)
Supplement: Supplementary file 1 — Additional file 1. The variations among descriptor values calculated by Mordred and other software programs. [file 13321_2018_258_MOESM1_ESM.docx]

**Additional file 1**

The variations among descriptor values calculated by Mordred and other software programs

A confirmation of the variations among descriptor values calculated by Mordred and other software programs (PaDEL-Descriptor, chemopy, and e-Dragon) are shown in Supplemental Table S2 in Additional file 2. Three molecules in different sizes were tested as examples: benzene as a small molecule, cyanidin as a middle-size molecule, and maitotoxin as a large molecule. All three molecules were inputted as an SDF file with 3D structures generated by Open Babel. Because the number of atoms of maitotoxin exceeded the limitation (maximum: 150 atoms) of e-Dragon, the descriptors of maitotoxin were not available for e-Dragon. Calculations of DetourMatrix, FMF, and WeightedPath of maitotoxin in PaDEL-Descriptor were also omitted because these descriptors cannot be calculated on account of their complexity. For Chemopy, only descriptors that are calculable without MOPAC were used.

Mordred provided the same descriptor values with all/any of the other software for most of descriptors. However, unique values were obtained by Mordred for several descriptors (Supplemental Table S2). The causes of these variations are listed in Supplemental Table S1.

Table S1. Causes of variations in descriptor values among Mordred and other descriptor calculation programs

| Descriptor No. | Causes for variations |
| --- | --- |
| 0002 | Mordred counts the sulfo group in maitotoxin as an acidic group, whereas PaDEL-Descriptor does not. |
| 0003 | Mordred counts O+ in cyanidin as a basic group, whereas PaDEL-Descriptor does not. |
| 0053-0061, 0152-0160, 0260-0268, 0368-0376, 0473-0480, 0569-0576, 0853, 0861 | The algorithm to calculate the intrinsic state differs between Mordred and PaDEL-Descriptor. In Mordred, it is coded to reproduce the values reported in the original paper. |
| 0233-0241, 0341-0349, 0449-0456, 0545-0553 | The results of the Gasteiger charge calculation are different between rdkit (on which Mordred depends) and CDK (on which PaDEL-Descriptor depends). |
| 0773 | In PaDEL-Descriptor, the bond between two rings in cyanidin is regarded as a multiple bond. Therefore, the value calculated by PaDEL-Descriptor is one smaller than that calculated by Mordred. |
| 0862-0904 | The values of surface area using numerical integration are different between Mordred and PaDEL-Descriptor. The surface area values calculated by Mordred are the same as the values calculated by another software, Pymol, under a 5% relative error. |
| 0647, 0660, 0673, 0686, 0725, 0738, 0924, | Constant value (709.78) is used when an overflow occurs in PaDEL-Descriptor. |
| 0014-0015, 0652-0653, 0665-0666, 0678-0679, 0691-0692, 0730-0731, 0743-0744, 0929-0930, | The formulations are different between Mordred and PaDEL-Descriptor. In Mordred, it is coded to reproduce the values reported in the original paper. |
